# Forecasting the morbidity and mortality of dengue fever in KSA: A time series analysis (2006–2016)

**DOI:** 10.1016/j.jtumed.2021.02.007

**Published:** 2021-03-18

**Authors:** Wajd A. Abualamah, Naeema A. Akbar, Hussain S. Banni, Mohammed A. Bafail

**Affiliations:** a Consultant in Department of Preventive Medicine and Public Health, Health Programs and Non-Communicable Diseases Management, Public Heath Directorate in Makkah, KSA; b Consultant in Department of Preventive Medicine and Public Health, Preventive Medicine and Residency Program Managment, Public Health Directorate in Jeddah, KSA; c Specialist in Department of Genetics, College of Medicine, Umm Al-Qura University, Makkah, KSA; d Researcher in Department of Physiology, College of Medicine, Umm Al-Qura University, Makkah, KSA

**Keywords:** حمى الضنك, تحليل السلاسل الزمنية, التنبؤ, الصحة العامة, العدوى الفيروسية, Dengue fever, Forecasting, Public health, Time series analysis, Viral infection

## Abstract

**Objectives:**

This study aimed to forecast the morbidity and mortality of dengue fever using a time series analysis from 2006 to 2016.

**Methods:**

Data were compiled from the Jeddah Dengue Fever Operations Room (RFOR) in a primary health care centre. A time series analysis was conducted for all confirmed cases of dengue fever between 2006 and 2016.

**Results:**

The results showed a significant seasonal association, particularly from May to September, and a time-varying behaviour. Air temperature was significantly associated with the incidence of dengue fever (p < 0.001) but was not correlated with its mortality. Similarly, relative humidity was not significantly associated with the incidence of dengue fever (*p* = 0.237).

**Conclusion:**

The strong seasonal association of dengue fever during May to September and its relation to air temperature should be communicated to all stakeholders. This will help improve the control interventions of dengue fever during periods of anticipated high incidence.

## الملخص

**أهداف البحث:**

هدفت هذه الدراسة إلى التنبؤ بمعدلات الإصابة بحمى الضنك ووفياتها باستخدام السلاسل الزمنية ٢٠٠٦-٢٠١٦.

**طرق البحث:**

تم تجميع البيانات من غرفة عمليات حمى الضنك بجدة في مركز الرعاية الصحية الأولية بمدائن الفهد. وتم إجراء تحليل السلاسل الزمنية لجميع الحالات المؤكدة لحمى الضنك خلال الفترة ٢٠٠٦-٢٠١٦.

**النتائج:**

أظهرت تحليل السلاسل الزمنية وقوع حالات حمى الضنك ووفيات موسمية قوية، خاصة من شهر مايو إلى شهر سبتمبر، وسلوك متغير بمرور الوقت. وارتبطت درجة حرارة الهواء بشكل رئيس بحدوث حمى الضنك ولكن لم تتوافق مع معدل الوفيات، في حين أن الرطوبة النسبية لم تكن مرتبطة بشكل كبير مع أي حالة من حالات حمى الضنك أو الوفيات.

**الاستنتاجات:**

بسبب حالات حمى الضنك والوفيات الموسمية القوية بناء على السلسلة الزمنية من شهر مايو إلى شهر سبتمبر وعلاقتها بدرجة حرارة الهواء، نوصي بنشر هذه البيانات على أصحاب المصلحة لتحسين تدخلات مكافحة حمى الضنك في الفترات التي يتوقع حدوث ارتفاع الإصابات فيها.

## Introduction

Dengue fever is a mosquito-borne viral infection that causes a flu-like symptoms and occasionally develops into a potentially lethal complication called severe dengue.^1^ Dengue is placed among the world's major public health concerns. Additionally, it is the most prevalent vector-borne disease with a wide geographic spread, and it can progress to serious and lethal forms.^2^ Epidemiologically, it is estimated that dengue infects more than 50 million people each year, and around two and a half billion people are at risk of infection worldwide.^3^ Although dengue mortality is 99% preventable, case fatality rates (CFR) far higher than 1% have been observed worldwide.^4^ According to estimations, dengue is responsible for 10,000 deaths in more than 125 countries.^5^ Recent statistics predict that 60% of the global population will be at risk for dengue fever in 2080.^6^ (see Figure 8)

Currently, dengue fever is considered as a main public health problem in several parts of the Kingdom of Saudi Arabia (KSA) (Makkah, Jeddah, Jazan, and Najran) with a dramatic increase in the number of cases reported every year.^7^ Moreover, the prevalence of dengue fever in Jeddah city was 47.8%.^8^ Current dengue prevention strategies are ineffective as they are reactive rather than anticipatory. Dengue surveillance is usually based on a passive system, that is, health professionals are mandated by law to report dengue cases to the health authorities. This system, however, does not sufficiently perceive the changes in incidence for an adequate response. Consequently, they may often be implemented late, thereby, reducing the opportunities for preventing transmission and controlling the epidemic.^9^

An early warning system is an essential tool for pre-epidemic preparedness and effectiveness of dengue control. In recent decades, weather variables such as temperature and rainfall have been widely studied as potential early warning tools to prevent climate-sensitive infectious diseases, such as Malaria, Dengue, and West Nile Virus.^10^ Statistical tools used in epidemiology to monitor and predict dengue and other infectious diseases have included time series analysis techniques such as autoregressive integrated moving average (ARIMA) models.^11^ The analysis of time series assumes that data represent consecutive measurements taken at equally spaced time intervals. Two primary goals of time series analysis are as follows: (a) identifying the nature of the phenomenon represented by the sequence of observations, and (b) forecasting (predicting future values of the time series variable).

The ARIMA methodology, which was developed by Box and Jenkins (1976), allows us not only to uncover the hidden patterns in the data, but also to generate forecasts. This methodology has gained enormous popularity in many areas and research practice confirms its power and flexibility. However, because of its power and flexibility, ARIMA is a complex technique; it is not easy to use, it requires considerable experience, and even though it often produces satisfactory results, those results depend on the researcher's level of expertise.^12^

Using time series analysis to analyse past trends of dengue fever outbreaks and incorporating this data into an appropriate statistical model to forecast future dengue fever outbreaks can be beneficial for improving current prevention and control measures. Considering all the factors that might influence the trends of outbreaks is also essential. Therefore, this study aims to forecast future dengue outbreaks in Jeddah city using time series analysis of data from 2006–2016.

## Materials and Methods

Monthly-confirmed dengue cases from January 2006 to December 2016 in Jeddah city were extracted from the dengue surveillance database (the old spreadsheet programme and the new Health Electronic Surveillance Network [HESN] programme) of the Dengue Fever Operational Room (DFOR) in the Public Health Directorate in Jeddah, KSA. The General Authority of Meteorology and Environmental protection centre in Jeddah was contacted, and they provided data pertaining to monthly temperature degrees and humidity levels in Jeddah from 2006–2016.

Data was prepared, coded, entered, and managed using Statistical Package for Social Sciences (SPSS) version 23 and assessed for normality and multicollinearity. Multiple seasonal autoregressive integrated moving average models (SARIMA) were tested. An appropriate model was defined, plotted, and used for prediction, followed by an analysis of multivariate regression for association with temperature and humidity.

## Results

**Figure 1:**
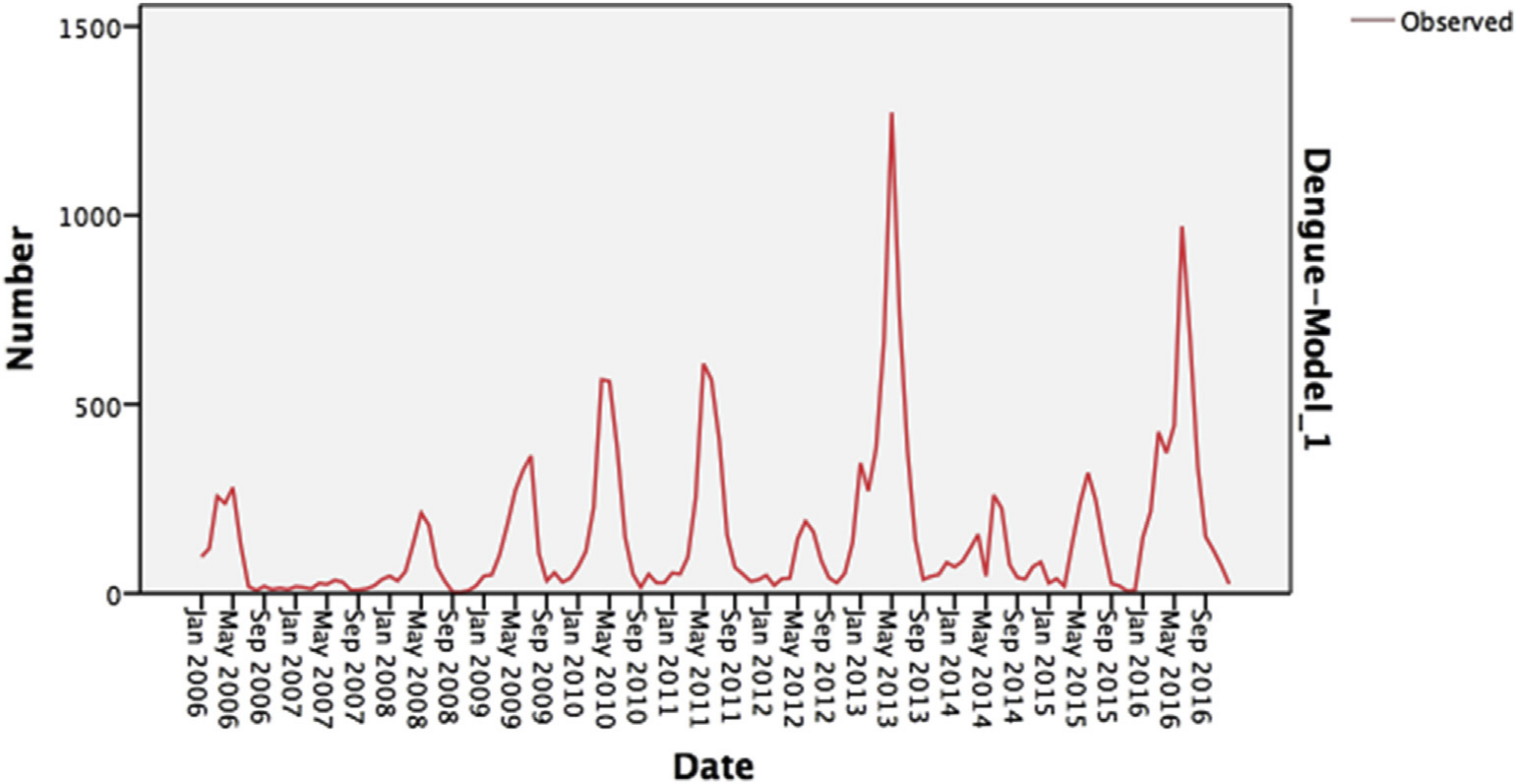
Monthly observed Dengue cases, KSA, 2006–2016, Showing data pertaining to time-varying behaviour and seasonality with high peaks from May to September each year.

The dengue time series consisted of the monthly numbers of all confirmed cases from 2006 to 2016. Visual inspection of the dengue time series showed a time-varying behaviour and a strong seasonality (Figure 1). The series appears to slowly gradually moves up and down with no obvious outliers. There were higher values presenting a trend, particularly in September, with the highest rate in 2013. This time-varying behaviour indicated by shifts in the trend over time suggested that the ARIMA models were appropriate.

**Figure 2:**
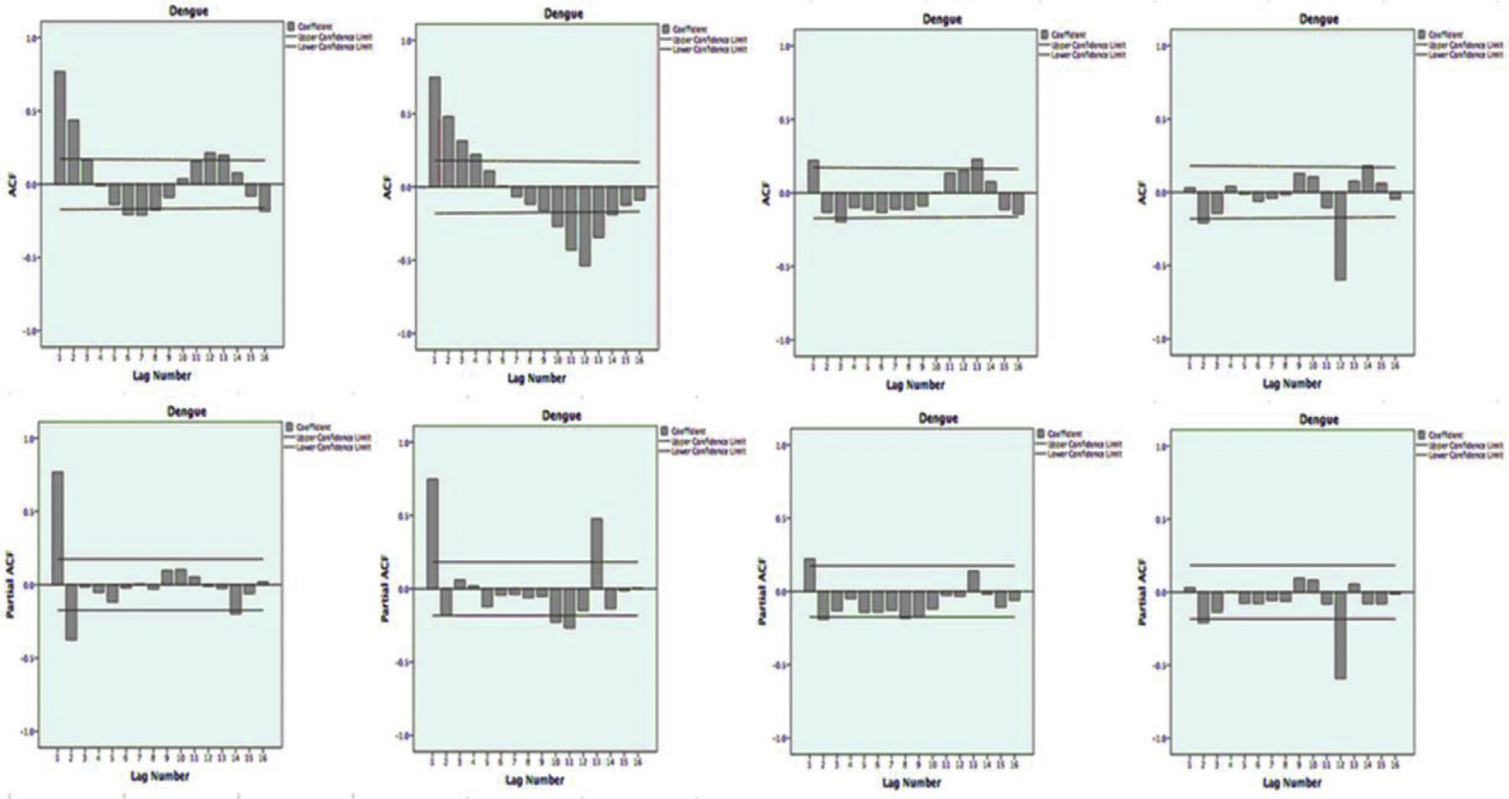
Autocorrelation and partial autocorrelation plots for Dengue time series; Autocorrelation function (ACF), Partial autocorrelation function (PACF) of data and transformed series. Footnote: (a) shows time series data without any difference; (b) shows transformed series with a seasonal difference (1, period 12); (c) shows transformed series with a non-seasonal difference^1^; (d) shows transformed series with both seasonal (1, period 12) and non-seasonal differences.^1^

First, we investigated the distributional properties of the data using time series' partial autocorrelation function (PACF) and autocorrelation function (ACF) related to the Bai and Perron model. The analysis resulted in stationary variables, suggesting that the change-point model could explain the intense changes in the dynamics, levels, and trends in the data (Figure 2).

The model identified the number and location (in time) of the breakpoints. Since adding more breakpoints will tend to result in lower errors in a regression context, a Bayesian information criterion (BIC) was used for the selection of the final model to ensure an optimal set of breakpoints for the selection. The number and location of the lagged values used in the model were chosen such that the residuals of the final regression were white noise or serially uncorrelated. The SARIMA (1,0,0) (0,1,1) was chosen to be used with the lowest BIC (9.240) and mean absolute percent error (MAPE) of 84.223. The parameters of the statistical model have a statistically significant effect in the time series with P-value > 0.05 and R2 of 72.1%.

**Figure 3:**
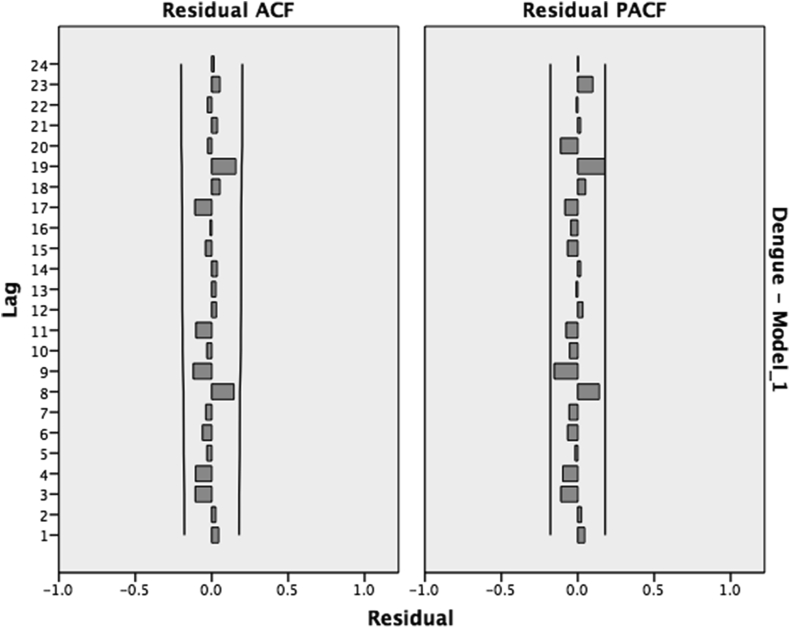
ACF and PACF of the Residuals,^1^ showing no autocorrelation in the dengue incidence time series.

**Figure 4:**
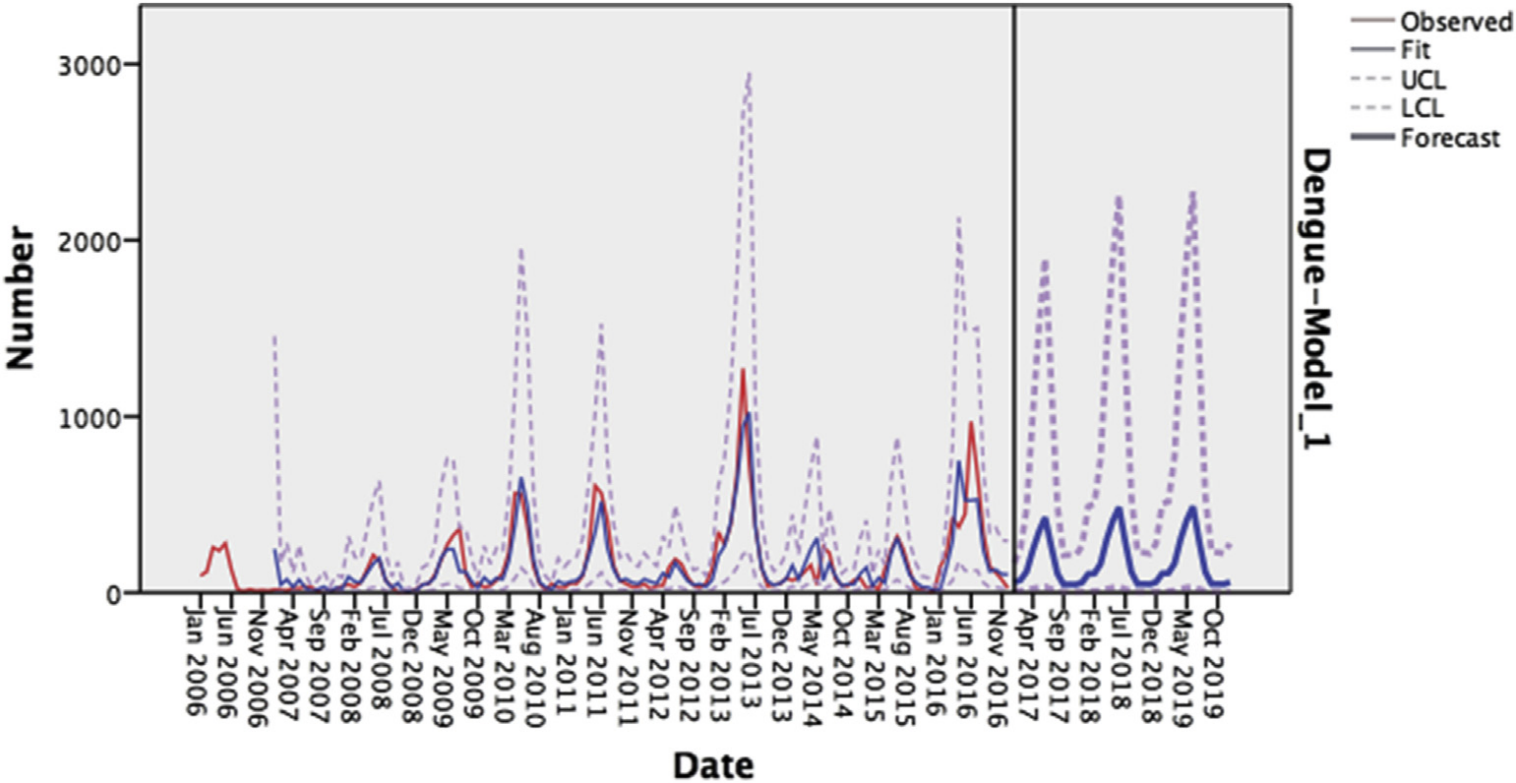
Monthly observed and forecasted dengue cases, KSA, 2006–2016; Observed = Dengue's series values, Fit = Simulate of the SARIMA (1,0,0) (0,1,1) model on our Observed Series, UCL = The highest possible value according to the model of SARIMA (1,0,0) (0,1,1), LCL = The lowest possible value according to the model of SARIMA (1,0,0) (0,1,1), Forecast = Forecast values of SARIMA (1,0,0) (0,1,1).

Ljung–Box Test was used to ensure the absence of time series’ autocorrelation. No autocorrelation could be found, with a P-value of 0.683. In this context, we have plotted the ACF & PACF of the residuals that showed no autocorrelation (Figure 3) and were also normally distributed. The final analysis model with observed and forecasted results can be seen in (Figure 4).

**Figure 5:**
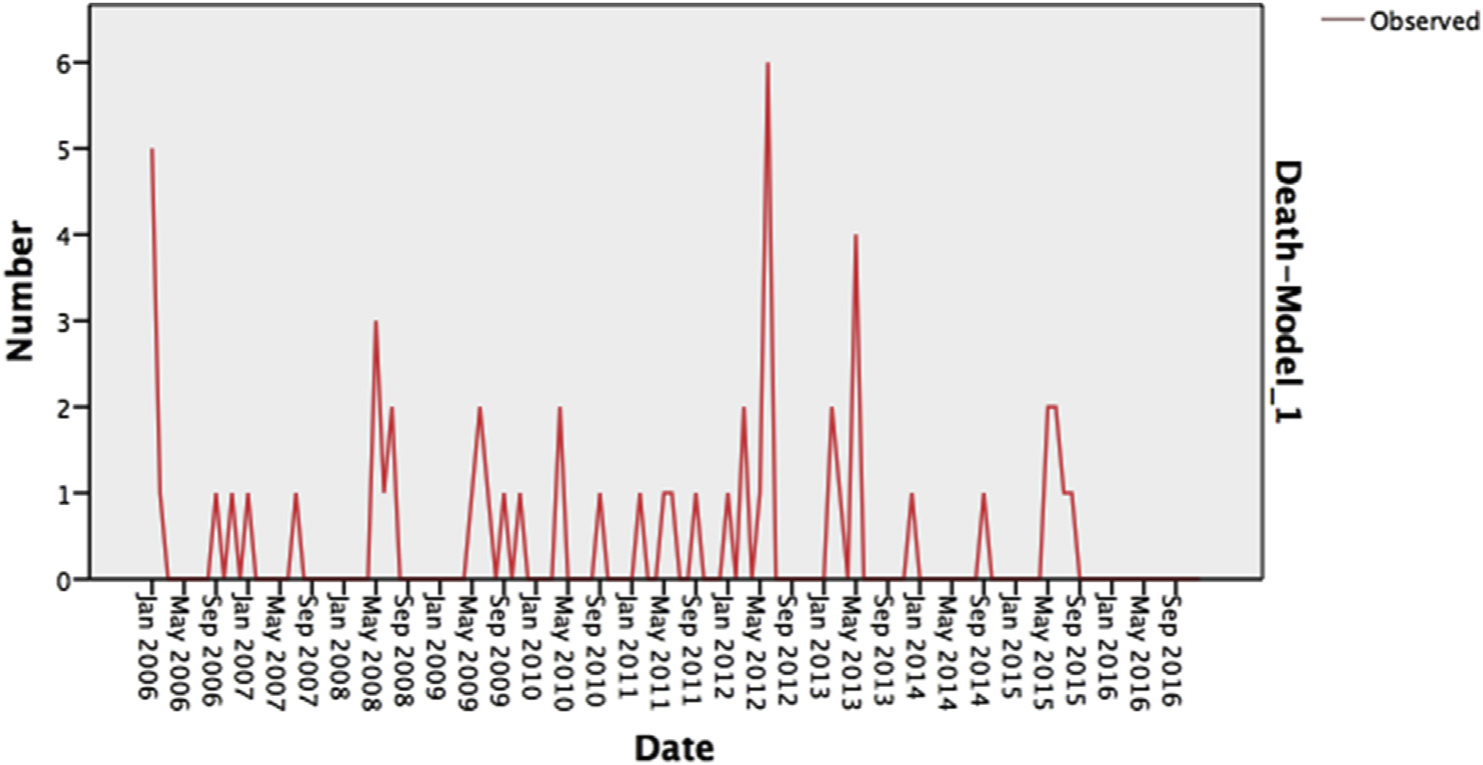
Monthly observed Dengue deaths, KSA, 2006–2016, Showing stochastic pattern.

The dengue time series consisted of the monthly numbers of all confirmed cases of deaths between 2006 and 2016. Visual inspection of the dengue time series showed a time-varying behaviour and a strong seasonality closely associated with a similar pattern of cases in the time series (Figure 5). The series showed a relatively stochastic pattern with no obvious outliers. There were higher values presenting a trend extending from May to September with the highest rate in 2012. This time-varying behaviour with the relative trend behaviour suggested that ARIMA models were initially appropriate.

**Figure 6:**
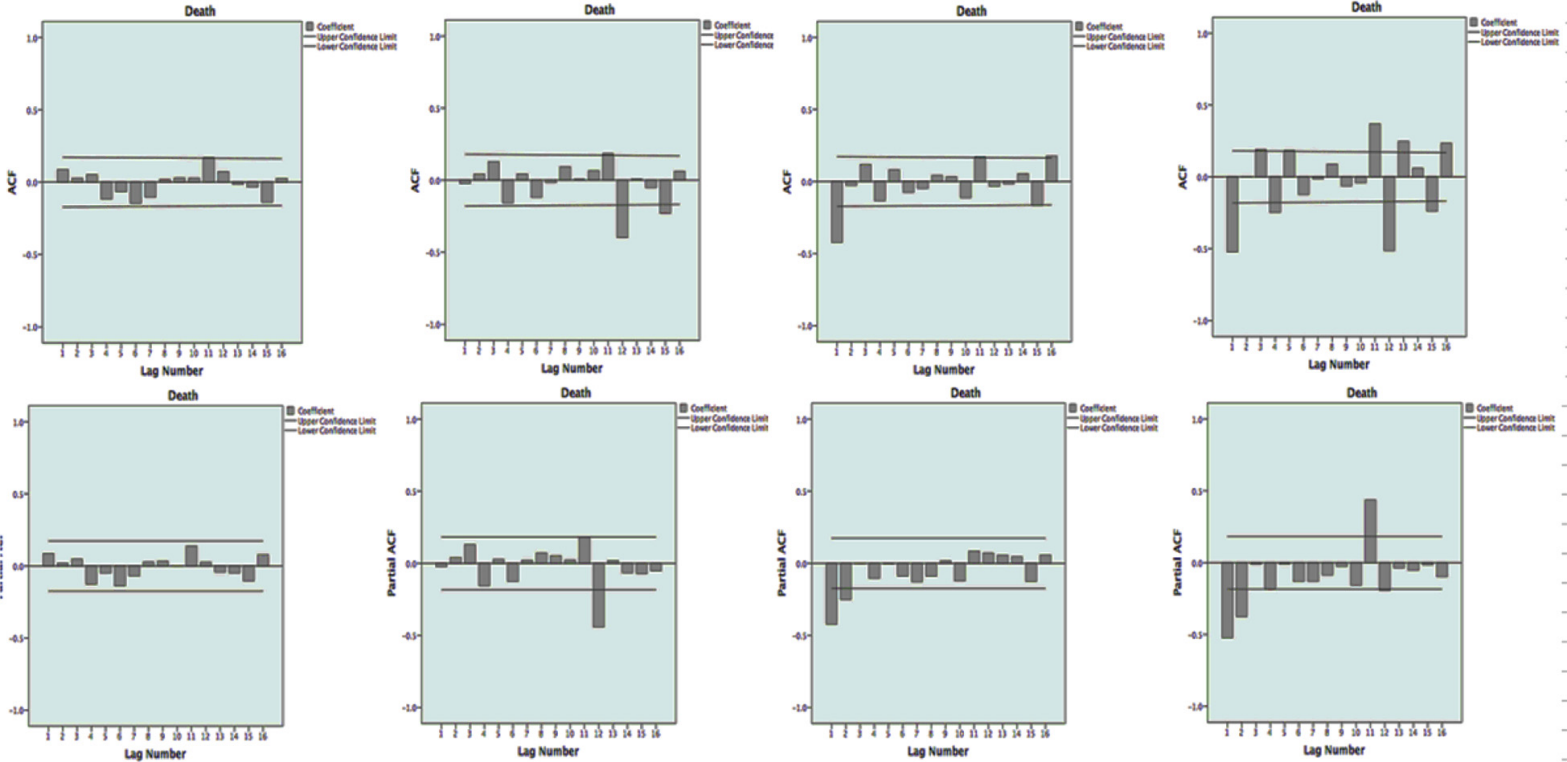
Autocorrelation and partial autocorrelation plots for Dengue time series; Autocorrelation function (ACF), Partial autocorrelation function (PACF) of data and transformed series. Footnote: (a) shows time series data without any difference; (b) shows transformed series with a seasonal difference (1, period 12); (c) shows transformed series with a non-seasonal difference^1^; (d) shows transformed series with both seasonal (1, period 12) and non-seasonal differences.^1^

**Figure 7:**
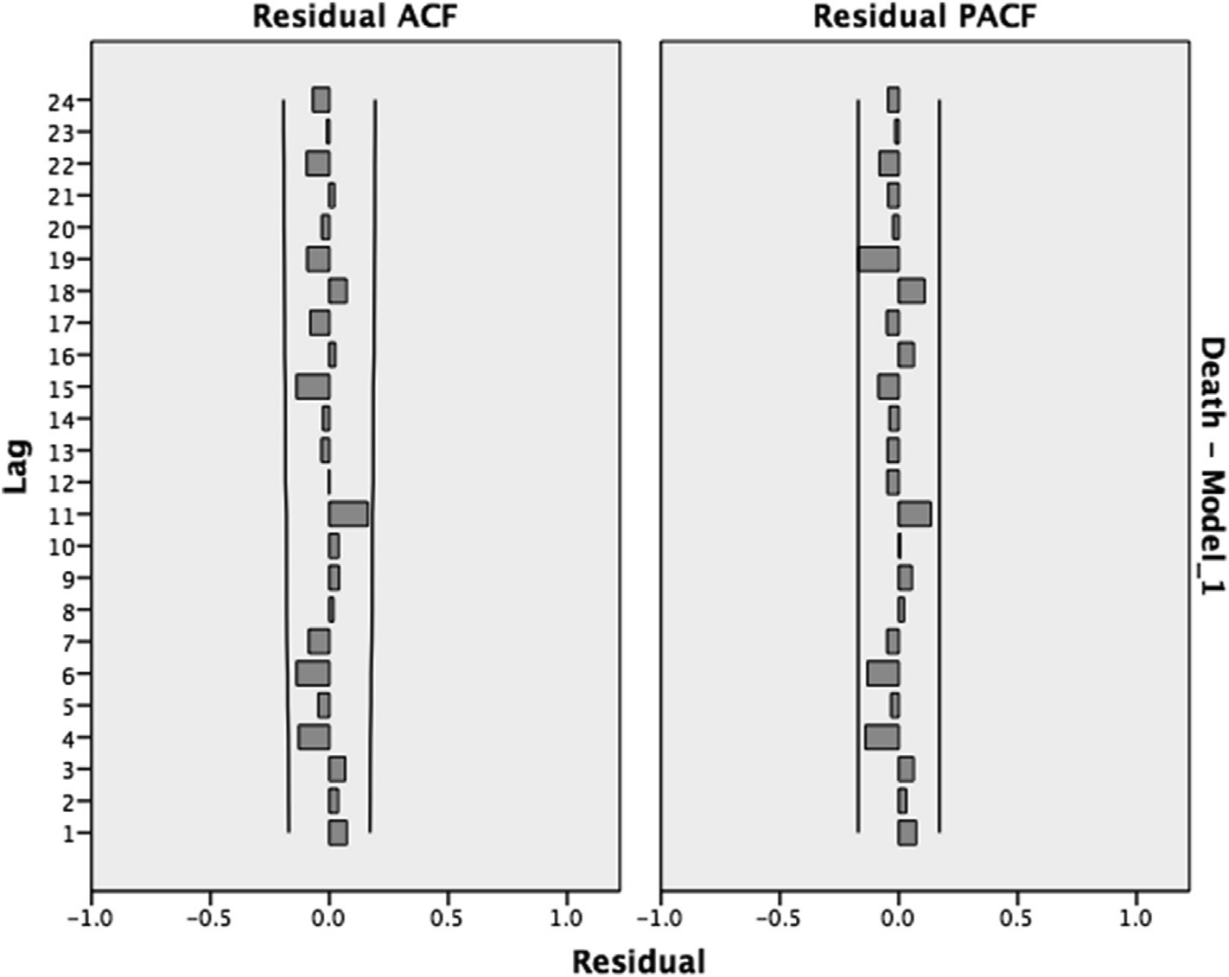
ACF and PACF of the Residuals,^2^ Showing no autocorrelation in the dengue death time series.

Similarly, the distributional properties of the data were investigated using time series’ PACF and ACF, suggesting that the change-point model could explain the changes in dynamics, levels, and trends in the data (Figure 6) (see Figure 7).

**Figure 8:**
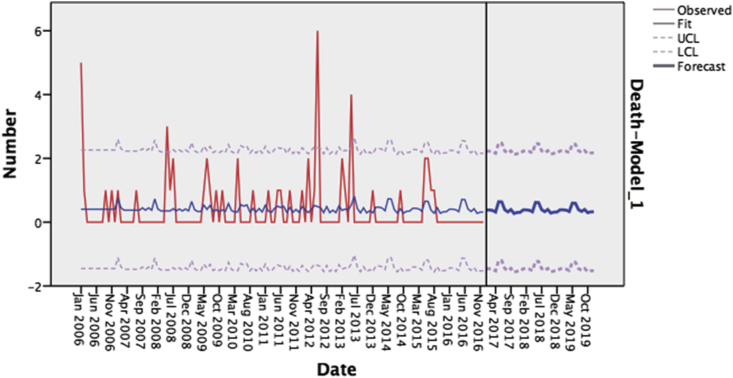
Monthly observed and forecasted dengue cases, KSA, 2006–2016; Observed = Dengue's series values, Fit = Simulate of the SARIMA (0,0,0) (1,0,1) model on our Observed Series, UCL = The highest possible value according to the model of SARIMA (0,0,0) (1,0,1) LCL = The lowest possible value according to the model of SARIMA (0,0,0) (1,0,1) Forecast = Forecast values of SARIMA (0,0,0) (1,0,1)

A time series regression analysis was performed to evaluate how well some factors can predict the trends in dengue's cases series. The predictors were air temperature and relative humidity, while changes and trends in dengue's series was the criterion variable. The results of regression showed a significant association (P > 0.001) with air temperature, and the correlation co-efficient was 0.0755. This association may explain trends and increased rates observed in Septembers of each year. Contrarily, there was no significant association (P = 0.231) with relative humidity (Table 1).

**Table 1:** Regression results for dengue's time series.

Dengue's Series	Coefficient	Standard Error	T-Statistic	P-Values
Air temperature	0.075515	0.009731	7.760	0.0001∗∗
Relative humidity	−0.006036	0.005012	−1.204	0.231

∗P-value > 0.05; ∗∗ P-value > 0.001.

Similarly, a time series regression analysis was performed to evaluate how well some factors can predict the trends in dengue's deaths series. The predictors were air temperature and relative humidity, while changes and trends in dengue's series was the criterion variable. The results of regression showed no significant association with neither air temperature or relative humidity with P-values of 0108 and 0.438, respectively (Table 2).

**Table 2:** Regression results for dengue's deaths series.

Death's Series	Coefficient	Standard Error	T-Statistic	P-Values
Air temperature	0.026928	0.016618	1.620	0.108
Relative humidity	−0.006664	0.008559	−0.779	0.438

∗P-value > 0.05; ∗∗ P-value > 0.001.

## Discussion

Dengue fever is a major public health concern in KSA. This is partly due to the dramatic increase in the number of cases reported every year. Unfortunately, there is a lack of preventive strategies. Health sector responds to the disease's occurrence in a reactive rather than an anticipatory manner. Eventually, this necessitates the presence of concrete figures that predict dengue fever's occurrence, morbidity, and mortality. This study aimed to assist the health authorities in adopting effective approaches to prevent the disease by forecasting dengue fever's incidence. To this end, two concise objectives were planned; to forecast the dengue fever morbidity and mortality using time series from 2006–2016 for the years 2017–2019 via a time series analysis consisting of monthly confirmed dengue fever cases from 2006–2016. The series showed that the disease had a strong seasonality with the highest rates occurring between May and September. The forecast showed peaks of incidence in the same months with the highest incidence predicted to be 489 cases in June 2019.

Similarly, researchers from Rajasthan developed a prediction model for dengue fever using time series data over the past decade to forecast monthly dengue fever/dengue haemorrhagic fever incidence for 2011. The SARIMA model was used for statistical modelling. Reported cases of dengue fever/dengue haemorrhagic from January 2001 to December 2010 showed a cyclical pattern with seasonal variation. The forecast for the year 2011 showed a seasonal peak in the month of October with an estimated 546 cases.^9^ Another study done in Ribeirão Preto, São Paulo State, Brazil, also used SARIMA to fit in a model of monthly reported cases of dengue fever from 2000–2008. The predicted values for the incidence for 2009 were extracted and compared with the results of the observed number of cases. The researchers found that the seasonal ARIMA model effectively and reliably predicts the number of dengue cases and is a useful tool for disease control and prevention.^13^

Regarding mortality, time series consisting of the monthly number of deaths of confirmed cases between 2006 and 2016 was used. The SARIMA model was less appropriate for this time series than that for the time series of cases. The series showed a relatively stochastic pattern with no obvious outliers. Higher values presented a trend extending from May to September with the highest rate of deaths in year 2012, and the most deaths from dengue were predicted to occur between May and June.

Some factors, namely air temperature and relative humidity, were studied to explore their impact on dengue fever morbidity and mortality time series. Air temperature was significantly associated with dengue using a time-series regression analysis (p < 0.001) with a correlation co-efficient of 0.0755. However, it did not seem to be significantly associated with dengue fever deaths. Contrarily, relative humidity had no significant impact, either on the disease morbidity or the mortality. Consistent with this study, a recent study conducted in Jeddah, KSA, in 2017 which aimed at modelling the association between dengue fever and some environmental factors stated that the maximum temperature was a predictor of the number of dengue fever cases in the city. However, researchers had also reported that the mean relative humidity is another predictor of the number of cases.^14^

Similarly, in 2014, a meta-analysis of data collected from 33 articles from six databases reported that temperature had a close association with the occurrence of dengue fever. The closest associations were observed between a mean temperature of 23.2–27.7 °C and dengue fever (OR 35.0% per 1 °C; 95% CI 18.3%–51.6%). Additionally, a minimum temperature of 18.1–24.2 °C and a maximum temperature of 28.0–34.5 °C were also associated with increased dengue fever transmission.^15^ Furthermore, a study conducted in Philippines that aimed at exploring the impact of temperature, relative humidity, and rainfall on dengue fever reported that all the three factors were significantly associated with the dengue fever epidemic.^16^ Consistent with these results, a study conducted in Argentina in 2012 stated that temperature alone, despite being useful in estimating the transmission risk, does not correlate with the distribution of dengue occurrence at the country scale on the national wide level.^17^

## Conclusion

To conclude, the time series analysis of dengue's incidence showed a strong seasonality, with higher incidence rates in May. The death analysis showed a similar series, with a relatively stochastic pattern and the highest values extending from May to September. Air temperature was significantly associated with the dengue disease incidence but not deaths, whereas relative humidity did not seem to be associated either with disease incidence, or mortality.

## Recommendations

Given this study's finding of anticipated incidence of dengue fever in the next three years, it is highly recommended that health care sectors adopt effective preventive measures to reduce incidence rates in the period between May and September. Additionally, more focus is required on the prevention of primary infection, which will in turn reduce severe dengue cases developing consequential to secondary infection. Data of predicted epidemics should be disseminated to the stakeholders at dengue fever control program and the Municipal of Jeddah for the advance intensification of control measures. Future longitudinal interventional studies are needed to study the impact of different preventive measures on dengue fever trends, morbidity, and mortality.

## Source of funding

This research did not receive any specific grant from funding agencies in the public, commercial, or not-for-profit sectors.

## Conflict of interest

The authors have no conflict of interest to declare.

## Ethical approval

This study was approved by the IRB of Directorate of Health Affairs- Jeddah with the registration number H-02-J-002 dated 11/04/1438H.

## Authors contributions

WAA and NA conceived and designed the study and obtained data. WAA, HSB, and MAB analysed and interpreted data. WAA, HSB, and MAB wrote the initial draft of the article. NA reviewed and edited the initial draft. All authors have critically reviewed and approved the final draft and are responsible for the content and similarity index of the manuscript.
